# SARS‐CoV‐2, HIV, and *Mycobacterium tuberculosis* triple co‐infection

**DOI:** 10.1002/ccr3.6018

**Published:** 2022-07-14

**Authors:** Marius Paulin Ngouanom Kuate, Felix Bongomin, Roland Ndip Ndip

**Affiliations:** ^1^ Department of Microbiology and Parasitology, Faculty of Science University of Buea Buea Cameroon; ^2^ Department of Medical Microbiology and Immunology, Faculty of Medicine Gulu University Gulu Uganda

**Keywords:** Cameroon, SARS‐CoV‐2, TB, triple coinfection

## Abstract

Tuberculosis (TB)‐related death has increased for the first time in a decade due to the coronavirus disease 2019 (COVID‐19), globally. People living with HIV (PLWHIV) might be at a higher risk of developing COVID‐19‐related complications. Herein, we describe the first case of a patient surviving from SARS‐CoV‐2‐TB‐HIV triple co‐infection in Cameroon. A 36‐year‐old Cameroonian woman presented at the emergency unit of the Jamot Hospital, Yaoundé with symptoms of anorexia, productive cough, weight loss, and fever. The SARS‐CoV‐2 rapid antigen test on nasopharyngeal sample was positive. Chest X‐ray showed bilateral parenchymal and tracheal calcifications most consistent with prior pulmonary histoplasmosis, varicella, or TB. She was tested HIV positive, and the sputum sample tested positive for TB on auramine staining. TB therapy (rifampicin, isoniazid, pyrazinamide, and ethambutol) and COVID‐19 treatment were initiated, and the symptoms improved after 2 weeks of treatment. The SARS‐CoV‐2 rapid antigen and real‐time polymerase chain reaction tests were negative after 2 weeks. She was discharged home on antiretroviral therapy and TB therapy. Coinfection with both TB, HIV, and SARS‐CoV‐2 may be common in Cameroon but not reported. The similar clinical features of COVID‐19 and TB usually lead to misdiagnosis. Early diagnosis and initiation of appropriate treatment improve outcome.

## Abstract 2

COVID‐19, TB, and HIV are infectious diseases of global public health concern. Triple Co‐infection with these diseases can create a diagnostic and therapeutic dilemma. We present a 36‐year‐old Cameroonian woman who survived SARS‐CoV‐2 – HIV‐TB triple co‐infection. Timely diagnosis and appropriate management of these infections may improve outcomes and prevent complications.

## 1 INTRODUCTION

The first case of the coronavirus disease‐2019 (COVID‐19) was reported in Cameroon on Friday, March 6, 2020 (MSF, 2020).^1^ From the first case to the June 03, 2022, the World Health Organization (WHO) has reported 119,947 confirmed cases of COVID‐19 with 1930 deaths in Cameroon.^2^ There are several complications associated with acute and post‐acute COVID‐19 syndromes. These sequelae include immunosuppression,^3^ superinfection, and co‐infections.^4^, ^5^ With an incidence of 174/100,000 populations, tuberculosis (TB) remains a public health problem in Cameroon.^6^ Deaths from TB have increased for the first time in a decade due to COVID‐19 in many countries of the world, including Cameroon.^7^ Several case reports have highlighted COVID‐19‐TB co‐infection but few have reported post‐COVID‐19‐TB^3^ and a triple COVID‐19—HIV—TB co‐infections.^8^ People living with HIV might be at higher risk of developing COVID‐19‐related complications and poor clinical outcomes due to various reasons such as a compromised immune system, presence of several co‐infections, and side effect of antiretroviral therapy (ART) medicines.^8^ The CD4^+^ T‐cell depletion associated with COVID‐19‐HIV co‐infection may significantly promote the development of active TB.^9^ Currently, there is no report on the outcome of SARS‐CoV‐2‐TB‐HIV triple co‐infection among Cameroonians. We describe the first case of a patient surviving from this mayhem in our clinical practice.

## 2 CASE PRESENTATION

A 36‐year‐old Cameroonian woman presented to the emergency unit of the Jamot Hospital, Yaoundé with symptoms of anorexia, productive cough, weight loss, and fever for a month. These were associated with frontal headaches and night sweats. She received home‐based management for COVID‐19 following a positive rapid antigen test for SARS‐CoV‐2. Her symptoms relapsed 2 weeks after completion of the COVID‐19 treatment, with recurrence of symptoms of fever, cough, with associated constitutional symptoms. However, the symptoms were much severe. She had no contributory past medical history.

Her oxygen saturation was 96%, and the blood pressure was 120 and 80 mmHg for systolic pressure and diastolic pressure, respectively. She had no conjunctival pallor, jaundice, cyanosis, peripheral lymphadenopathy, or digital clubbing. Chest examination revealed bilateral versicular breath sounds with added coarse crepitations. Other systemic examinations were unremarkable.

A possible diagnosis of SARS‐COV‐2 persistence or re‐infection was made. Differential diagnosis included post‐acute COVID‐19 syndrome, malaria, sepsis, bacterial pneumonia, TB, histoplasmosis, and other HIV‐related opportunistic infection.

**FIGURE 1:**
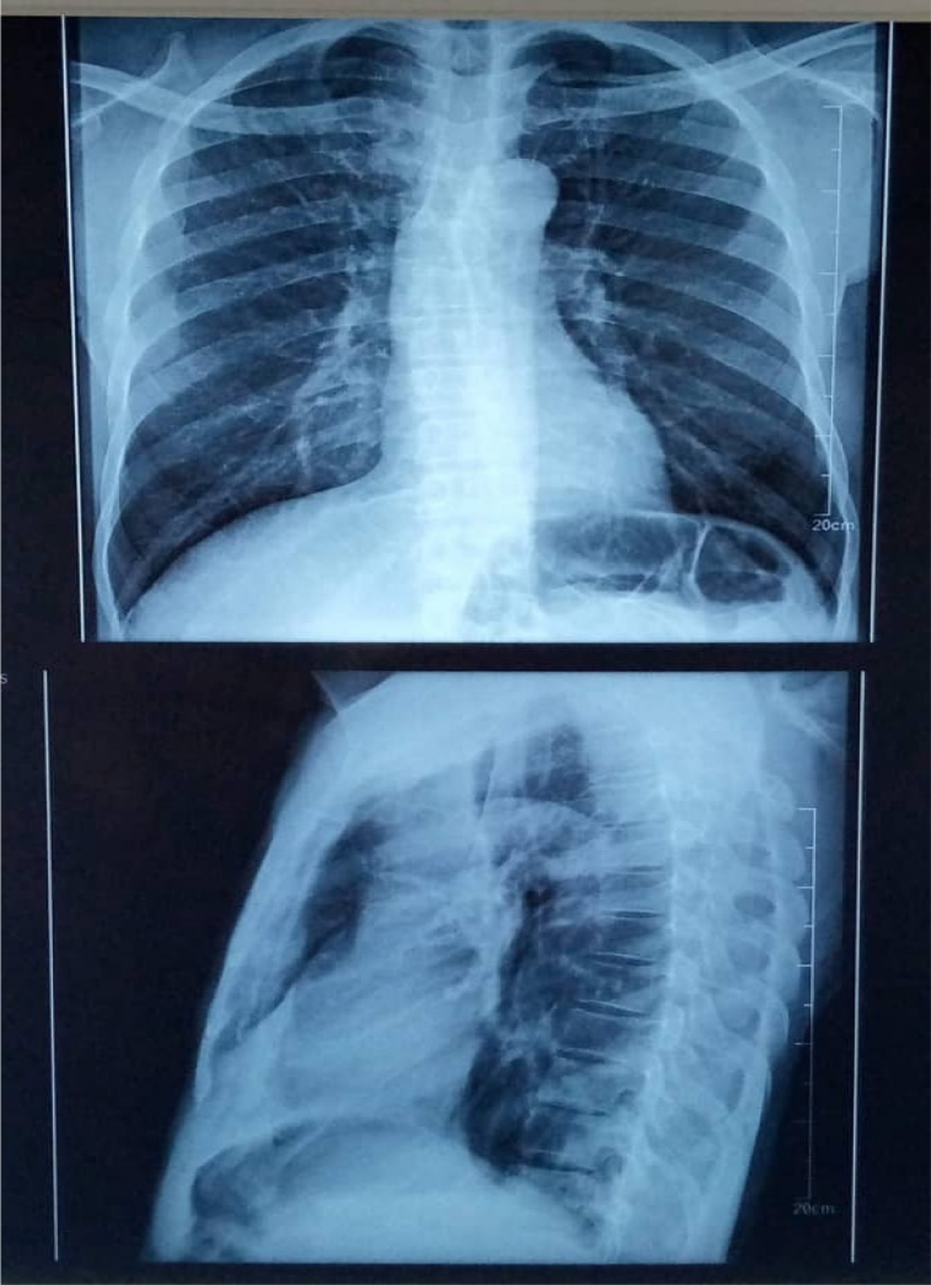
Chest X‐ray result showing Bilateral dots of calcification and Small areas of patchy calcification posteriorly in the left base

**FIGURE 2:**
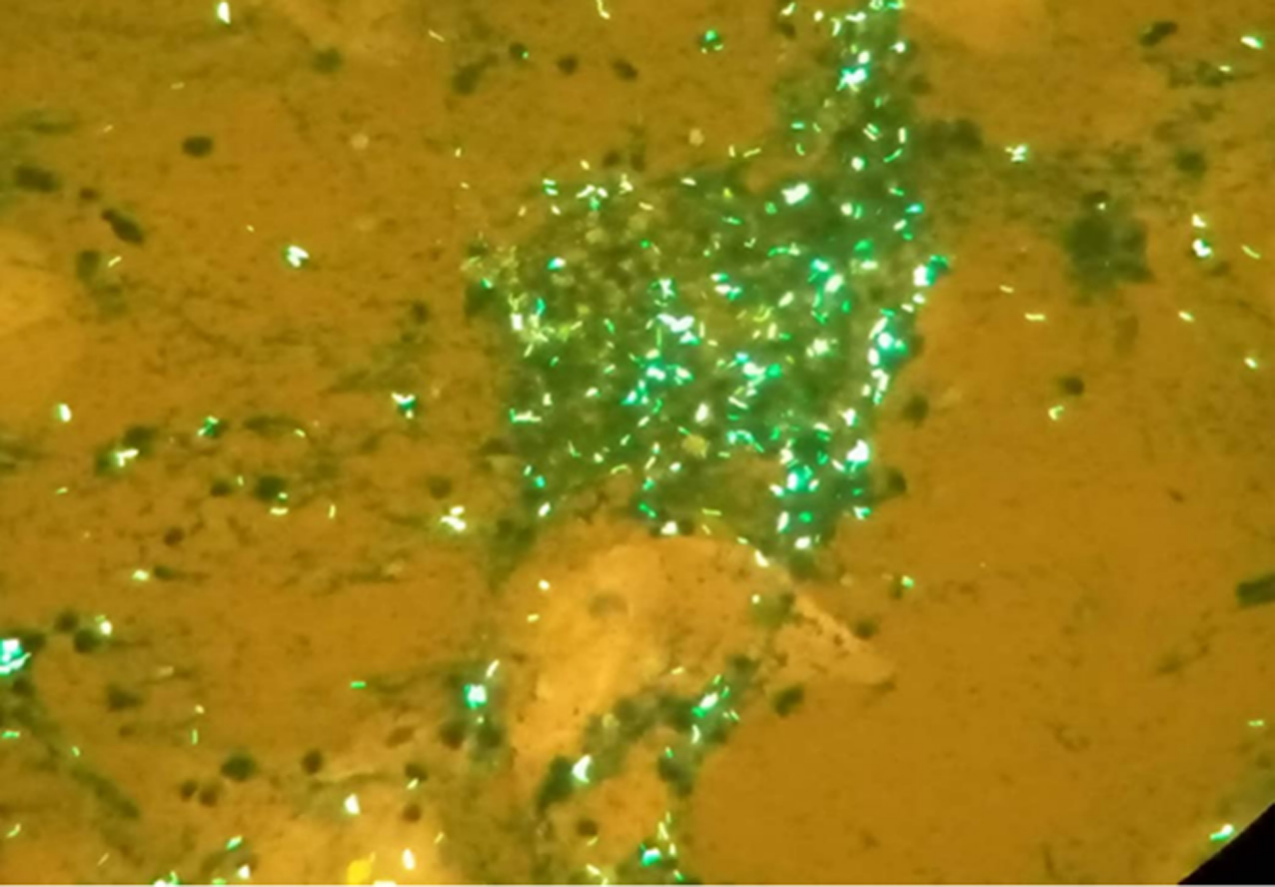
Auramine TB staining showing fluorescent bacilli

Both SARS‐CoV‐2 antigen and real‐time polymerase chain reaction (RT PCR) tests were positive. Chest X‐ray performed showed bilateral dots of calcification most consistent with prior histoplasmosis, varicella, or tuberculous infection; enlargement of the left hilum with calcified bronchi and also some tracheal calcification; small areas of patchy calcification posteriorly in the left base, visible on the lateral view, normal bones, and heart outline (Figure 1). The sputum sample sent to the laboratory for TB auramine staining was positive (Figure 2). Her HIV test was also positive.

A standard TB therapy regimen was initiated, that is, RHEZ (rifampicin, isoniazid, pyrazinamide, and ethambutol). After 8 days of both COVID‐19 treatment and TB treatment, we had amelioration of the symptoms. After 2 weeks of treatment, both SARS‐CoV‐2 rapid antigen tests and RT‐PCR for COVID‐19 came out negative, the protocol for COVID‐19 was discontinued. In the 3rd week, she was initiated on antiretroviral therapy (ART) and allowed home.

## 3 DISCUSSION

In this report, we described a case of TB‐SARS‐CoV‐2 co‐infection in a young Cameroonian woman newly diagnosed with HIV, without previous ART experience. Literature on COVID‐19 and moreover co‐infection with chronic infectious diseases, such as HIV and TB, is rare in Cameroon. However, a study revealed that male gender, diabetes mellitus, and hypertension are the most important risk factors for severe COVID‐19 and mortality in Cameroon.^10^

HIV and TB interaction with COVID‐19 is poorly understood. However, it is recognized that persons living with HIV are immunocompromised and are more susceptible to TB and, possibly COVID‐19.^11^ About 27% of TB cases reported in 2019 in Cameroon occurred among persons living with HIV.^12^ People living with chronic infectious diseases such as HIV and TB are more likely to experience poor outcomes from COVID‐19.^8^ Severe cases of COVID‐19 manifest mainly as fever, dry cough, dyspnea, headache, rhinorrhoea, and rarely as hemoptysis.^13^, ^14^

The diagnosis and management of COVID‐19 is more challenging when co‐infected with TB due to the similarity of their clinical features or when there are existing lung lesions due to previous lung diseases. The main chest imaging findings for severe COVID‐19 are pulmonary opacities with a diffuse distribution, even with complete opacification of both lungs.^15^ Though our patient viral load and CD4+ T‐cell count was unknown, it has been shown that HIV patient with low CD4+ T‐cell count, and high viral load have a high risk of acute respiratory distress syndrome. Moreover, HIV patient have threefold higher chance of dying from COVID‐19 than those without HIV.^8^ There is no doubt that co‐infection could be a significant promoter to the final mortality of COVID‐19 patients; therefore, the early recognition and managements of bacterial co‐infection seem rather indispensable to decrease the mortality of COVID‐19 patient as much as possible.^16^

## 4 CONCLUSION

There are several complications associated with acute and post‐acute COVID‐19 syndromes. Immunosuppression associated with COVID‐19‐HIV co‐infection may significantly promote the development of active TB. The co‐infection with both TB, HIV, and SARS‐CoV‐2 may be common in Cameroon but not reported and published. Moreover, the similar clinical features of COVID‐19 and TB usually lead to misdiagnosis. The immunocompromising disease, HIV, can triple the risk of fatality when co‐infected with COVID‐19. However, this case shows that early diagnosis and management improves outcomes in co‐infected individuals.

## AUTHOR CONTRIBUTION

Marius P. Ngouanom Kuate involved in data collection, wrote the first draft, and read and approved the final draft. Felix Bongomin revised the first draft, and read and approved the final draft. Roland Ndip Ndip supervised the work, and read and approved the final draft.

## CONFLICT OF INTEREST

None.

## CONSENT

Written informed consent was obtained from the patient to publish this report in accordance with the journal's patient consent policy.

## Data Availability

The data that support the findings of this study are available from the corresponding author upon reasonable request.
